# A Surgical Perspective on Targeted Therapy of Hepatocellular Carcinoma

**DOI:** 10.3390/diseases3040221

**Published:** 2015-09-29

**Authors:** Claire Faltermeier, Ronald W. Busuttil, Ali Zarrinpar

**Affiliations:** Dumont-UCLA Transplant Center, Division of Liver and Pancreas Transplantation, Department of Surgery, David Geffen School of Medicine, University of California at Los Angeles, Los Angeles, CA 90095, USA; E-Mails: CFaltermeier@mednet.ucla.edu (C.F.); rbusuttil@mednet.ucla.edu (R.W.B.)

**Keywords:** hepatocellular carcinoma, liver resection, liver transplantation, locoregional therapy, targeted therapies, molecular signatures, biomarkers, immunotherapy

## Abstract

Hepatocellular carcinoma (HCC), the second leading cause of cancer deaths worldwide, is difficult to treat and highly lethal. Since HCC is predominantly diagnosed in patients with cirrhosis, treatment planning must consider both the severity of liver disease and tumor burden. To minimize the impact to the patient while treating the tumor, techniques have been developed to target HCC. Anatomical targeting by surgical resection or locoregional therapies is generally reserved for patients with preserved liver function and minimal to moderate tumor burden. Patients with decompensated cirrhosis and small tumors are optimal candidates for liver transplantation, which offers the best chance of long-term survival. Yet, only 20%–30% of patients have disease amenable to anatomical targeting. For the majority of patients with advanced HCC, chemotherapy is used to target the tumor biology. Despite these treatment options, the five-year survival of patients in the United States with HCC is only 16%. In this review we provide a comprehensive overview of current approaches to target HCC. We also discuss emerging diagnostic and prognostic biomarkers, novel therapeutic targets identified by recent genomic profiling studies, and potential applications of immunotherapy in the treatment of HCC.

## 1. Introduction

Hepatocellular carcinoma (HCC) is the sixth most common cancer and the second leading cause of cancer deaths worldwide [1,2]. Agents causing chronic liver disease and eventually cirrhosis are risk factors for developing HCC [3]. These include infection with hepatitis B virus (HBV) and hepatitis C virus (HCV), alcoholic liver disease, and nonalcoholic fatty liver disease [4]. Other predisposing factors with lower prevalence are hereditary hemochromatosis, alpha1-antitrypsin deficiency, autoimmune hepatitis, and Wilson’s disease [4]. In the United States, the incidence of HCC has tripled over the past decade, but the five-year survival rate of 12% has not changed [4]. The poor prognosis associated with HCC is due to multiple factors, some of which include: (1) occurrence usually in the background of severe liver disease; (2) lack of effective therapeutics for advanced disease; and (3) aggressive and heterogeneous tumor biology [5,6,7]. Cures are possible if HCC is diagnosed early and treated with resection, liver transplantation, and/or ablation [8]. In this review we will discuss different approaches (Table 1) to targeting HCC including targeting the tumor anatomically and targeting the tumor biology. We will also review emerging biomarkers and molecular targets which promise to improve early detection and treatment of late-stage disease.

### Article Selection

Representative articles were selected from references found by searching Pubmed for the following key words: Targeting HCC anatomically section: “HCC”, “HCC and surgical resection”, “HCC and anatomical resection”, “liver transplantation and HCC”, “living-donor liver transplantation”, “percutaneous ethanol injection”, “radiofrequency ablation”, “microwave ablation”, “transarterial chemoembolization”, “transarterial radioembolization”, “cryoablation”, “irreversible electroporation”, “laser ablation”, and “high-intensity focused ultrasound;” Targeting HCC tumor biology section: “sorafenib and HCC”, and “kinase inhibitors and HCC”; Future directions section: “serum biomarkers and HCC”, “AFP and HCC”, “DKK1 and HCC”, “microRNAs and HCC”, “metabolomics and HCC”, “gene-expression signatures and HCC”, “immunotherapy and HCC”, “JX-594”, “CTLA-4 and HCC”, and “glypican-3”.

## 2. Targeting HCC Anatomically: Resection, Liver Transplantation, and Locoregional Therapies

### 2.1. Resection

The goal of surgical resection is to remove the tumor while preserving as much liver function as possible to prevent postoperative liver failure [9]. Surgical resection is one of the most effective treatments for patients with HCC, yet more than 70% of patients are ineligible [10]. Factors contributing to ineligibility include: extrahepatic metastasis, multiple and bilobar tumors, involvement of the main bile duct, or presence of tumor thrombus in the main portal vein and/or vena cava [11].

The selection of appropriate candidates for resection not only involves the assessment of feasibility of complete tumor resection, but also the remnant liver function and a prediction of how much liver volume can be safely removed. Ideal candidates for resection have HCC without cirrhosis, usually in the context of HBV infection. However, such cases are rare and account for only 5% of patients in Western countries [8]. Most patients with cirrhosis have a high morbidity and mortality following anesthesia and surgery [12]. The use of Child-Pugh classification, model of end-stage liver disease (MELD), or clearance of indocyanine green to predict the risk of postoperative complications can be informative, but has not yet been proven to be reliable [13,14]. The presence of portal hypertension assessed by a hepatic venous pressure gradient ≥10 mmHg or suggested by the presence of a platelet count below 100,000/µL [3], splenomegaly or history of varices, has been reported to be a significant predictor of postoperative hepatic decompensation [8,14].

In patients with normal liver function, it is usually considered safe to remove up to 70% of the total liver volume (TLV) [9]. However, the regenerative capacity of the liver is significantly decreased in patients with cirrhosis, and at least 50% of TLV should be preserved [9]. For cases in which the predicted remnant liver volume is below safety limits, preoperative portal vein embolization can stimulate liver hypertrophy to increase future remnant liver size [15].

Operative considerations include the extent of resection (limited *vs.* anatomical) and method (open *vs.* laparoscopic). Since HCC is assumed to metastasize to other sites via the portal vein, Makuuchi *et al.* introduced the practice of “anatomical resection” or tumor removal with its portal tributaries by segmentectomy [16]. For tumors between 2 to 5 cm sizes, anatomical resections achieve significantly better disease-free and overall survival than limited resections [9,17,18]. For patients with tumors <2 cm size and poor liver function, limited resections are preferred since small tumors have a low risk of dissemination [9,11]. When considering open *vs.* laparoscopic resections, multiple studies have shown long-term oncologic outcomes are similar. However, laparoscopic resections are associated with reduced blood loss, postoperative complications, and length of hospital stay [19]. Despite these advantages, laparoscopic resections have numerous technical challenges and should be performed only by experienced surgeons [20].

In a study of 6785 cirrhotic patients treated by liver resection, the Liver Cancer Study Group in Japan reported that short-term survival rates are good (one-, three-year 88%, 69%), but long-term survival rates are poor (five-, 10-year 53%, 28%) [21]. High rates of tumor recurrence (>80% five years after resection) contribute to poor long-term survival [22]. Preventing recurrence with neoadjuvant or adjuvant therapies has had limited success [8]. Randomized controlled trials (RCTs) using preoperative hepatic artery chemoembolization or adjuvant systemic chemotherapy have not improved overall survival [23,24,25,26]. In fact, Ono and colleagues reported that systemic chemotherapy actually correlated with a lower disease-free and overall survival [27]. Immunotherapy, internal radiation, and differentiation therapy (retinoids) may lengthen disease-free survival after resection, but these therapies require testing in large RCTs [8,23,28,29,30,31,32]. The oral multikinase inhibitor sorafenib has proven benefit in the treatment of non-resectable HCC. Whether sorafenib can reduce recurrence rates is under investigation in the Sorafenib as Adjuvant Treatment in Recurrence of Hepatocellular Carcinoma (STORM) trial [33].

**Table 1:** Current treatment approaches for patients with HCC.

Intervention	Indication & Patient Tumor Characteristics	Patient Liver Function	Clinical Outcomes	Disadvantages	Emerging Treatment Advancements
Resection	-Localized -Single tumor -Resection should preserve >50% TLV [9] -Bridging tx to transplant ^1^ [1]	No portal hypertension [8,14]	-Recurrence: 80% within five years [22] -Five-year survival: 53% [21]	High surgical morbidity in patients with cirrhosis [12]	-Biomarkers to predict recurrence [34,35,36] -Postoperative adjuvant sorafenib [33] -Laparoscopic resection [19,20]
Liver transplantation	-Localized -Single tumor <5 cm or 2–3 tumors ≤3 cm (Milan criteria) [37]	Decompensated cirrhosis ok (Child-Pugh C)	-Recurrence: ~18% at one year [38] -Five-year survival: 70%–80% if within Milan criteria [37,39]	-Shortage of donor organs -Long waiting time [40]	-Nomograms and biomarkers to predict recurrence [41,42,43,44,45,46] -Post-transplant adjuvant sorafenib [47] -Expansion of Milan criteria [48,49] LDLT, and use of ECD livers [7,8,50]
Percutaneous ethanol injection (PEI)	Localized Tumors <3 cm [8]	Preserved liver function (Child-Pugh A) [8]	-Recurrence: 43% for tumors >3 cm at 2 years [51] -Five-year survival: 28%–40% (single tumor <3 cm) [52]	Multiple treatment sessions required	Use decreasing in US, as RCTs have shown RFA is superior to PEI for tumors >2 cm [53,54,55]
Radiofrequency ablation (RFA)/Microwave ablation (MWA)	-Localized, unresectable -Tumors <4 cm [7] -Bridging tx to transplant [56]	Preserved liver function (Child-Pugh A)	-Recurrence: 50% within three years [54,57] -Five-year survival: 33%–40% (≤3.5 cm) [57]	-More adverse events *vs.* PEI [58,59] -RFA is less effective for highly vascular tumors [60]	Emerging ablation methods have potential to treat pts with advanced liver disease and tumors near vital structures [61,62,63,64]
Transarterial chemoembolization (TACE)/Transarterial radioembolization (TARE)	-Localized, multifocal, unresectable [65] -Tumors >4 cm [7] TARE for pts w/ portal vein thrombosis [66,67,68] -Bridging tx to transplant [56]	Preserved liver function (Child-Pugh A) [65]	Two-year survival: 63% (Child-Pugh A) [69,70]	-Low CR rate (6%) [71] -Post-embolization syndrome in 60%–80% of pts [72]	-TACE w/ drug eluting beads [73] -TACE + sorafenib [74,75]
Sorafenib	-Metastatic, unresectable -Any size -Vascular invasion ok [76]	Preserved liver function (Child-Pugh A) [77]	-Radiological progression: 75% of pts within six months [77] -One-year survival: 44% [77]	-No CR or PR [77] -Unclear efficacy in pts with poor liver function (Child-Pugh B or C) [77]	-Combination therapies (sorafenib + RFA, TACE, liver transplant) [75,78,79] -Molecular analysis of tumors to predict tx response [80,81]

^1^: Emerging indication, but not widely used. Abbreviations: TLV, total liver volume; year, year; tx, treatment; pts, patients; w/, with; CR, complete response; PR, partial response; LDLT, living donor liver transplantation; ECD, expanded criteria donor.

### 2.2. Liver Transplantation

Liver transplantation (LT) is one of the most effective therapeutic options for patients with HCC as it removes both macroscopic and microscopic tumors and treats the underlying liver disease [11]. Before 1996, LT was reserved for patients with unresectable large or multifocal HCC. The results of such LTs were disappointing due to the high rate of recurrent disease in the new allograft and poor survival [82,83]. A landmark publication by Mazzaferro *et al.* established the Milan criteria by demonstrating that patients who have either one tumor <5 cm in diameter or 2–3 tumors each with a diameter of <3 cm have lower rates of disease recurrence [37]. Furthermore, patients transplanted within the Milan criteria have a five-year survival (70%–80%) similar to patients transplanted for non-HCC indications [37,39].

Due to the scarcity of donor organs, selecting patients who will benefit most from LT has promoted strict adherence to the Milan criteria. In the United States, LT waitlist priority (currently starting MELD equal to 22 with additional points every three months) is only given to HCC patients within the Milan/T2 staging criteria [84,85,86,87]. However, multiple centers have reported acceptable outcomes when transplanting patients outside of the Milan criteria [48]. The University of California, San Francisco (UCSF) group transplanted patients with single tumors <6.5 cm or 2–3 tumors <4.5 cm with a total diameter <8 cm (UCSF criteria) and reported excellent survival [48]. At our institution we found similar five-year survival rates for patients transplanted within the Milan *vs.* UCSF criteria (79% *vs.* 64% *p* = 0.061) [49]. Nevertheless, the question still remains whether expanded criteria which results in slightly lower survival rates can justify the use of scarce donor organs [88]. Bruix and colleagues proposed transplantation for HCC patients should only be considered when patients’ five-year expected survival is at least 50% [89]. Yet, when comparing the survival benefit of patients transplanted outside the Milan criteria to the harm inflicted on other patients on the waiting list, Volk and colleagues proposed a five-year expected survival cutoff at 61% [90].

While the time from listing to transplantation varies based on geographic location, many HCC patients experience tumor progression and drop out from the waiting list. The UCSF group showed the probability of dropout at six, 12, and 24 months to be 7.3%, 25.3%, and 43.6% [40]. To lower dropout rates, bridging treatments such as radiofrequency ablation, transarterial chemoembolization, or percutaneous ethanol injections are recommended, especially if waiting time is expected to exceed six months [91,92,93]. In fact, waiting more than six months on the transplant list drastically improved post-transplant survival as it seemed to select out the tumors with poor biology [94]. Surgical resection prior to transplantation is also an option. Belghiti *et al.* demonstrated that the resection of tumors within the Milan criteria did not increase transplantation surgery risk, nor reduce post-transplant survival [95].

The most effective strategy to reduce waitlist dropout is to expand the donor pool. Use of marginal or extended criteria livers (non-heart beating donors, split livers, domino transplants from patients with amyloidosis, and advanced aged-donors) has expanded the donor pool but not enough to significantly reduce wait times [8]. Living donor liver transplantation (LDLT) is considered a feasible alternative to cadaveric liver transplantation since it eliminates the need to wait and patients with HCC are often suitable candidates for small allografts [7]. Survival rates for patients undergoing LDLT are similar to cadaveric donor transplant [50]. When following UCSF criteria, a multicenter study in Korea showed three-year survival for LDLT was 91% compared 88% for cadaveric donors [96]. Despite these favorable outcomes for the recipient, the risk to the living donor is not negligible. Postoperative complications arise in 20%–40% of donors and the risk of mortality is 0.3%–0.5% [50,97].

HCC recurrence occurs in 8%–18% of patients after transplantation and is associated with a median survival of only nine months [38,49,98]. Identifying adjuvant therapies to prevent recurrence is an urgent need. Studies investigating systemic cytotoxic chemotherapy after transplantation have shown conflicting results, but the current consensus is that cytotoxic chemotherapy has failed to show major improvements in disease-free or overall survival [85,99,100,101]. Switching post-transplant immunosuppression from calcineurin inhibitors (CNI) to mTOR inhibitors may lower rates of recurrence [102]. CNIs can activate pro-tumorigenic pathways while mTOR inhibitors have both anti-proliferative and anti-angiogenic effects [103,104,105]. Encouraging results from multiple uncontrolled clinical studies have shown patients on sirolimus-based immunosuppressive protocols have a higher disease-free and overall survival compared to patients on CNI-based protocols [106,107,108,109]. However, a recent retrospective report comparing tumor recurrence and survival of >1000 patients transplanted for HCC found no differences between sirolimus users *vs.* non-users [110]. Perhaps the most promising adjuvant option is sorafenib [77,111]. At UCLA, in a case-controlled match study, Saab *et al.* showed sorafenib was well tolerated in transplant recipients and extended the disease-free survival (sorafenib 85.7% *vs.* control 57.1%) [47]. Based on these results, we started the POST trial, a phase II randomized, blinded, multicenter prospective study (NCT01624285) to determine if sorafenib is effective in preventing recurrence in high-risk HCC patients’ post-transplant.

Since most patients within the Milan/UNOS criteria undergoing transplant will not have tumor recurrence, prognostic indicators are needed to determine who will benefit from active surveillance and adjuvant therapy. Tumor size and pathologic features including differentiation, presence of vascular invasion, and nuclear beta-catenin localization have been shown to be independent predictors of recurrence [41,102,112,113]. An evaluation of molecular signatures based on tumor expression of 20 metastasis-associated microRNAs [42], a comparison of microRNA expression between tumor and adjacent benign tissue [43], or the tumor expression of five genes (HN1, RAN, RAMP3, KRT19 and TAF9) [44] can also predict prognosis. Levels of the serum markers alpha-fetoprotein (AFP) and des-gamma carboxyprothrombin (DCP) correlate with tumor recurrence post-transplant [45,114]. Still, no single pathological or serum marker has proven >90% sensitivity and specificity in predicting recurrence, and multiple groups have proposed a combination of markers can better predict prognosis [46,115]. Recently, we developed a nomogram incorporating laboratory values (pre-transplant AFP, total cholesterol, neutrophil-to-lymphocyte ratio), pathologic features (nuclear grade, vascular invasion), radiographic tumor size, and response to downstaging therapy that showed excellent accuracy in predicting recurrence in 865 liver transplant recipients [46].

### 2.3. Non-Surgical Targeting: Locoregional Therapies

Anatomical targeting of HCC by locoregional therapy is the next best treatment option for patients who are ineligible for surgical resection, liver transplant, or those who require downstaging or bridging therapy prior to transplantation. There is a growing list of locoregional approaches which can be divided into ablative and transarterial therapies. Local ablation involves killing tumor cells by chemical (ethanol, acetic acid) or thermal means (e.g., radiofrequency, microwave frequency, cryotherapy, laser) [56]. Transarterial therapies are characterized by arterial injection of therapeutic agents into the tumor followed by the occlusion of tumor blood supply [56].

#### 2.3.1. Percutaneous Ethanol Injection

Percutaneous ethanol injection (PEI) is considered a safe, inexpensive, and effective ablative therapy for small HCC tumors [8,60,116]. Under ultrasound or CT guidance, absolute ethanol is injected into the tumor causing tumor coagulation necrosis [116]. Depending on the tumor size, injections are repeated weekly for six to eight weeks. Other agents, such as acetic acid, have been tried, but outcomes are not superior to ethanol injections [117,118]. In Child class A patients with tumors under 5 cm, PEI results in five-year survival rates up to 50% [51,52,116,119]. However, high rates of tumor recurrence (40% at two years for tumors >3 cm) and multiple treatment sessions required have limited the use of PEI in current clinical practice [120].

#### 2.3.2. Radiofrequency Ablation

Radiofrequency ablation (RFA) has replaced PEI at most centers due to superior efficacy and shorter treatment times. RFA is performed percutaneously by advancing an electrode into the tumor and delivering energy in the form of radiowaves. The energy induces the rapid vibration of ions in the tissue resulting in frictional heat causing thermal destruction (coagulative necrosis) of the tumor [60]. Five RCTs have compared the efficacy of RFA to PEI. All studies reported lower rates of tumor recurrence in RFA-treated patients. Three studies demonstrated RFA confers a survival benefit for patients with tumors >2 cm [53,54,55,121,122]. However, drawbacks of RFA include higher cost, increased incidence of adverse events, and limitations depending on tumor location. In a meta-analysis of four RCT complications such as peritoneal bleeding, tumor cell seeding, or intrahepatic abscesses were observed in 4.3% of RFA-treated patients *vs.* in 2.7% of PEI-treated patients [123]. Since the mechanism of RFA-induced necrosis is dependent on heat, the cooling effect of blood flow makes RFA less effective for highly vascular HCC tumors or tumors adjacent to blood vessels [60]. RFA is also contraindicated for subcapsular tumors and tumors in close proximity to the gallbladder. Clinical experience has shown these locations are associated with incomplete tumor ablation and risk of damage to adjacent structures [58,59,116].

#### 2.3.3. Microwave Ablation

Similar to RFA, microwave ablation (MWA) destroys tumor tissue with heat. Instead of agitating ions within tissue, MWA uses high frequency electromagnetic radiation to heat intracellular water molecules resulting in coagulative tumor necrosis [56]. MWA is advantageous compared to RFA for several reasons including the ability to achieve higher intra-tumoral temperatures, ablate larger volumes, and it is not constrained by proximity to blood supply [56,116,124]. Still, there is no conclusive evidence showing MWA is more effective than RFA [57,125,126]. Several recent studies proposed that MWA may be an effective treatment for medium-to-large HCC tumors (>4 cm) [127,128,129,130]. Additional RCTs are needed to determine which patients are most likely to benefit from MWA.

#### 2.3.4. Transarterial Chemoembolization

As HCC tumors grow (>2 cm) they become more vascularized and receive blood flow almost entirely from the hepatic artery [72]. Transarterial chemoembolization (TACE) takes advantage of this dependence on the hepatic artery and is the treatment of choice for tumors greater than 4 cm or multifocal HCCs [7]. Performed under angiography, TACE involves advancement of a catheter into the hepatic artery, injection of a chemotherapy emulsion (usually doxorubicin and lipiodol), followed by arterial embolization, most frequently with 1 mm gelfoam cubes [8]. The survival benefit of TACE has been debated due to mixed results in several RCTs [69,70,131,132]. However, a meta-analysis confirmed TACE, in comparison to conservative management, increased the two-year survival of patients with multifocal HCC, preserved liver function, and lack of extrahepatic spread and vascular invasion [133]. In contrast, TACE has failed to show survival benefit in patients with decompensated cirrhosis (Child-Pugh B/C) [134,135].

While TACE can lengthen survival in select patients, ultimately 70%–80% will die due to tumor progression [70]. One way to potentially improve the efficacy of TACE is to increase the concentration and duration of the chemotherapy that reaches the tumor with drug-eluting beads (DEBs). In a European multi-center phase II prospective randomized trial (PRECISION V) the DEB-TACE group had fewer adverse events and a higher complete response rate (CR) and objective response rates (ORR) compared to the TACE control group (CR 27% *vs.* 22% and ORR 52% *vs.* 44%) [73]. Another promising approach is combining DEB-TACE with anti-angiogenic agents. Instead of undergoing necrosis, resistant tumors respond to TACE-induced hypoxia by increasing production of pro-angiogenesis factors such as VEGF [135,136]. Several trials have investigated the synergy of DEB-TACE and sorafenib, which inhibits VEGFR [74]. While initial efficacy data is promising, questions remain regarding the optimal dosing schedule (sequential, interrupted, or continuous) of sorafenib [75]. Results from two ongoing phase III RCTs (SPACE study and ECOG 1208), each with different dosing schedules, will provide insight to this question.

#### 2.3.5. Transarterial Radioembolization

HCC is a radiosensitive tumor; however, external beam radiotherapy is contraindicated for patients with cirrhosis due the risk of radiation-induced hepatitis [137]. To reduce the radiation of normal liver parenchyma and selectively target HCC, a type of brachytherapy involving the injection of yttrium-90 microspheres into the tumor-feeding vessels of the hepatic artery is used. Unlike TACE, transarterial radioembolization (TARE) maintains the patency of the hepatic artery and is suitable for patients with portal-vein thrombosis, a contraindication for TACE [66]. In a retrospective study by Sangro *et al.*, TARE was associated with a median survival of 15.4–16.6 months in patients who were poor candidates for TACE (bilobar, bulky disease, multiple >5 tumors) [67,68]. Patients with portal vein thrombosis were also treated in the study, and while survival was poor, it was comparable to survival associated with standard-of-care chemotherapy [66,67,68]. Several matched patient cohort studies have demonstrated TARE in comparison to TACE results in increased OS, relative response rate, and greater effectiveness in downstaging prior to transplantation [138,139,140]. RCTs comparing TACE and TARE are ongoing and will hopefully provide consensus if and when TARE is superior to TACE.

### 2.4. Emerging Ablation Methods

#### 2.4.1. Cryoablation

Although less commonly used, cryoablation, irreversible electroporation, laser ablation, and high-intensity focused ultrasound have important advantages to consider when optimizing treatment for HCC patients (Table 2). Cryoablation is performed percutaneously by advancing cryoprobes into the tumor and using either liquid nitrogen or argon gas to rapidly freeze tumor tissue [61]. Freezing to −35 °C induces the formation of ice crystals which damage cell membranes and organelles, leading to cell death [61]. Two recent analyses, one RCT and one prospective study, reported that the safety profile and outcomes of cyroablation were similar to RFA and MWA in the treatment of HCCs <2 cm [141,142]. However, cryoablation was superior in achieving local tumor control for tumors >2 cm. While additional large trials are needed, cryoablation may become a first-line ablative therapy for medium-sized tumors.

**Table 2:** Emerging ablation methods.

Method	Advantages	Status of Clinical Studies	Efficacy
Cryoablation	Less painful and may be optimal ablation method for medium-sized tumors [61]	One RCT and multiple prospective studies [141,142]	Similar to RFA/MWA for tumors <2 cm, superior efficacy for tumors >2 cm [141,142]
Irreversible electroporation (IRE)	Suitable for tumors adjacent to blood vessels [143,144]	Prospective studies only [62]	No studies yet comparing IRE to other methods
Laser ablation	Low cost (70% < RFA) and technical ease [145]	RCTs [63,145]	Equivalent to RFA for tumors <4 cm [63,145]
High intensity focused ultrasound (HIFU)	Option for patients with decompensated cirrhosis (Child-Pugh C), completely extracorporeal, effective even if tumor is near major hepatic vessels [64,146,147]	Prospective studies only [146,148]	Effective as a bridging therapy to transplantation [146]

#### 2.4.2. Irreversible Electroporation

Irreversible electroporation (IRE) is emerging as an excellent approach for HCC tumors near vital structures. A percutaneous approach is used to position electrodes around the tumor which give multiple, millisecond high voltage (>500 V/cm) electrical pulses. This results in the irreversible breakdown of cell membranes and hemorrhagic necrosis [62]. Since IRE is not temperature-dependent, it can be used on tumors adjacent to blood vessels. Importantly, IRE has been shown to effectively ablate tumors within the liver hilum while preserving the structure and functionality of the hepatic artery, portal vein, and bile duct [62,143,144]. There are no clinical trials yet comparing IRE to other ablation techniques; however, prospective studies suggest outcomes are similar [149]. For patients with tumors <3 cm in locations that would be too risky to treat with other ablative techniques, IRE seems to be a promising approach.

#### 2.4.3. Laser Ablation

Another percutaneous approach is laser ablation (LA). LA uses thin fibers (~300 μm) to deliver near-infrared light to tumor tissue. Upon absorption, the light is converted to heat, resulting in hyperthermia-induced cell death [63]. Retrospective studies have shown LA is safe and, more recently, a RCT showed LA was equivalent to RFA in achieving complete ablation of tumors <4 cm [145,150]. One significant advantage of LA is its low cost (70% less expensive compared RFA) [145].

#### 2.4.4. High-Intensity Focused Ultrasound

Unlike other modalities, high-intensity focused ultrasound (HIFU) is completely extracorporeal and lacks the risks of bleeding and tumor seeding with the direct puncture of tumors. Under MRI guidance, ultrasound beams are focused to the depth of the tumor and the absorption of acoustic energy generates heat, which results in tumor coagulation necrosis [64]. A group at the University of Hong Kong has published multiple small prospective studies suggesting that, in addition to being non-invasive, HIFU is safe for patients even with advanced liver disease (Child-Pugh class C) and may be considered as a bridging treatment to transplantation [146,148] (Table 3).

**Table 3:** Comparison of ablation methods.

Patient Tumor Characteristics	PEI	RFA	MWA	TACE	TARE	CRYO	IRE	Laser	HIFU
Small tumor <2 cm	+	+	+	−	−	+	+	±	+
Medium tumor <4 cm	−	+	+	+	+	+	+	+	+
Large tumor >4 cm	−	−	−	+	+	−	−	−	−
Multifocal	−	±	±	+	+	±	±	±	±
Near vascular structures	−	−	+	−	−	−	+	−	+
Decompensated cirrhosis	−	−	−	−	−	−	−	−	+
Portal vein thrombosis	−	−	−	−	+	−	−	−	−

Abbreviations: +, recommended; −, not recommended/no evidence supporting use; ±, may be considered; PEI, percutaneous ethanol injection; RFA, radiofrequency ablation; MWA, microwave ablation; TACE, transarterial chemoembolization; TARE, transarterial radioembolization; CRYO, cryoablation; IRE, irreversible electroporation; Laser, laser ablation; HIFU, high intensity focused ultrasound.

## 3. Targeting HCC Tumor Biology

### Chemotherapy

More than 70% of patients with HCC present with advanced disease and are poor candidates for anatomical targeting (resection, transplantation, or locoregional therapies) [33]. Historically targeting HCC tumor biology with chemotherapy has been ineffective. HCC is inherently chemoresistant and the altered drug metabolism of cirrhotic livers makes many chemotherapies highly toxic [78]. However, in 2007, sorafenib, a multi-kinase inhibitor targeting VEGFR, PDGFR-B, c-kit, FLT3, and cRAF, became the first systemic therapy approved for advanced HCC (Child-Pugh A/B, unresectable, metastatic or with vascular invasion) [79]. In the SHARP study, a phase III, randomized, double-blind, placebo-controlled trial, patients treated with sorafenib *vs.* placebo had a significantly improved OS (10.7 *vs.* 7.9 months *p* < 0.001) [77]. The side effect profile of sorafenib was tolerable and included hand-foot skin reactions (8%), diarrhea (8%), and fatigue (3%) [77]. Due to the impressive response of HCC to sorafenib, multiple studies are investigating the efficacy of sorafenib in other stages of disease. In particular, trials are under way using sorafenib following liver transplantation, resection, TACE, and TARE [76,78,151].

The FDA-approval of sorafenib represents a great leap for the treatment of advanced HCC; yet therapeutics are needed for patients with intolerance or acquired resistance to sorafenib. Over the past five years, phase III RCTs have compared the efficacy of multiple other kinase inhibitors. In the first-line setting, anti-angiogenic agents (sunitinib, brivanib, and linifanib) targeting VEGFR and PDGFR or combinations of sorafenib with anti-proliferative agents (erlotinib) targeting EGFR failed to show superiority to single-agent sorafenib with respect to overall survival [152,153,154,155]. Likewise, in the second-line setting, the VEGFR/FGFR inhibitor brivanib and the mTOR inhibitor everolimus did not meet primary endpoints of demonstrating superiority in overall survival when compared to placebo [156,157].

The disappointing results of numerous phase III RCTs using kinase inhibitors could be due to wrong molecular targets, high toxicities in patients with underlying liver cirrhosis, or poor patient selection [80]. Based on trials using brivanib and erlotinib, one might assume that inhibiting FGFR or EGFR pathways would have low antitumoral potency in HCC. On the contrary, FGFR and EGFR might still be effective targets, but only in selected patients whose tumors exhibit activation of such pathways. A recent phase II trial using tivantinib, a c-MET tyrosine kinase inhibitor, in the second-line setting provides rationale for treatment stratification based on tumor biomarkers [158]. Initial analyses showed the time to progression was similar between the tivantinib *vs.* placebo-treated group (1.6 *vs.* 1.4 months). However, when the tivantinib-treated group was stratified based on immunohistochemical detection of c-MET expression, patients with high c-MET-expressing tumors had a significantly longer time to progression (2.7 months). To enable better patient selection, predictive biomarkers are being identified for other therapies. Examples include high AFP levels as an indicator of response to the VEGFR2 inhibitor, ramucirumab, and genomic amplifications of VEGFA or FGF3/4 as markers of sensitivity to sorafenib [81,159,160].

## 4. Future Directions: Improving the Targeting of HCC

**Figure 1:**
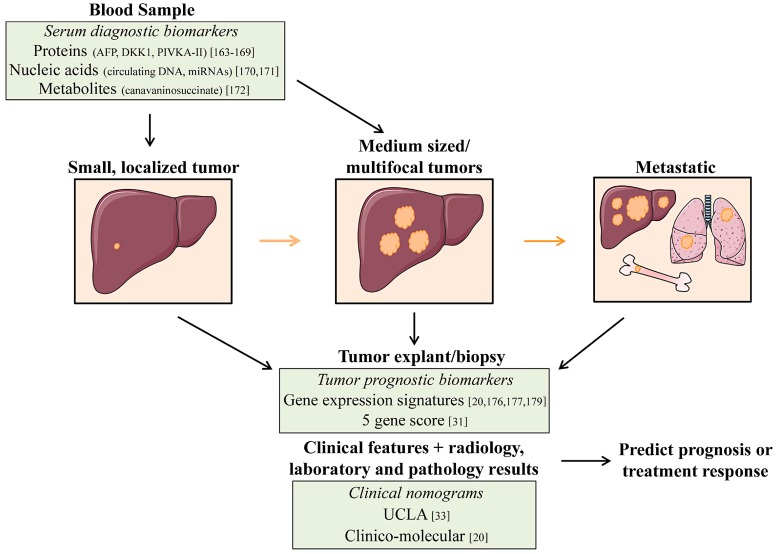
Proposed integration of biomarkers into HCC treatment. (**Left**) Few patients present with HCC tumors amenable to curative therapies, so emphasis has been placed on biomarkers to detect early-stage HCCs. Proteins, nucleic acids, and metabolites released by the tumor into circulation can provide a non-invasive method of early detection. (**Middle, Right**) Most treatment decisions for HCC patients are currently based on tumor size and liver function. However, these parameters cannot accurately predict optimal therapies for all patients and, in particular, those with metastatic disease. Tumor gene expression signatures can characterize tumor biology and aid in predicting prognosis and treatments.

Despite numerous treatment options, HCC is still one of the most lethal cancers worldwide. Anatomical targeting of HCC is the most effective treatment option; however, less than 30% of patients are eligible due to advanced tumor stage at diagnosis. Yet, even if eligible for surgical and/or locoregional therapies, the high recurrence rate of HCC impedes long-term survival. Although sorafenib was recently approved for advanced HCC, the median survival of patients is only one year. Hence, there is a critical need to: (1) improve methods for early detection so that more patients are eligible for curative therapies; (2) identify prognostic markers to improve patient selection and surveillance postsurgical or locoregional therapies; and (3) investigate the molecular mechanisms driving HCC progression to identify new therapeutic targets (Figure 1).

### 4.1. Diagnostic Biomarkers

Diagnosis of HCC without pathologic confirmation is currently based on serum AFP and imaging (ultrasound, MRI, CT). AFP levels are associated with tumor size, and only about two-thirds of HCC patients with tumors <3 cm will have elevated AFP levels [161]. Moreover, the specificity of AFP for HCC is low since elevated AFP is also detected in the serum of patients with cirrhosis and hepatitis [10]. MRI and CT are expensive, and ultrasound, while specific, is highly operator-dependent and has poor sensitivity in detecting HCC in patients with underlying cirrhosis [162]. Due to risks associated with biopsies in patients with cirrhosis and the need for cost-effective tests, research has been focused on identifying proteins, nucleic acids, and metabolites that could enable HCC diagnosis through serological testing.

The search for serum protein markers for early detection of HCC has identified numerous promising candidates including: des-gamma-carboxy-prothrombin (PIVKA-II), glypican-3, the ratio of glycosylated AFP (L3 fraction) to total AFP, alpha fucosidase, Dickkopf-1 (DKK1), and osteopontin [163,164,165,166,167,168]. The majority of these candidates have yet to show superiority to AFP. However, a recent retrospective trial comparing serum DKK1 to AFP found DKK1 could enhance the diagnostic accuracy of HCC [167]. Serum DKK1 was not only able to distinguish HCC from chronic liver disease, but could also detect HCC in early-stage patients who had normal AFP levels [167]. While the data for DKK1 is intriguing, additional studies are needed before it can be accepted as a valid marker for HCC screening. Rather than reliance on just one serum marker, a combinations of markers (*i.e.*, AFP, osteopontin, and DKK1) may provide superior sensitivity and specificity in detecting HCC [169].

The detection of circulating nucleic acids, particularly microRNAs (miRNAs), has the potential to be used in HCC diagnosis. miRNAs are small, non-coding RNAs which negatively regulate gene expression and have important roles in hepatocarcinogenesis [170]. In addition to their presence in HCC tumor tissue, some miRNAs can be found in systemic circulation, providing rationale to investigate their use in HCC diagnosis. In a large cohort study of 934 patients (healthy, chronic HBV, cirrhosis, and HBV-related HCC), a panel of seven miRNAs (miR-122, miR-192, miR-21, miR-223, miR26a, miR-27a, and miR-801) had a sensitivity of 83% and specificity of 94% in diagnosing HCC [171].

Nomograms combining clinical, radiology, laboratory, and pathology results can also predict HCC recurrence after resection or transplant. These predictions can guide adjuvant therapy and tumor surveillance.

In comparison to hepatocytes in cirrhotic or normal livers, metabolic profiling of HCC cells has identified alterations in pathways associated with phospholipid, fatty acid, and bile acid metabolism [172,173]. These differences have instigated the search for cancer-associated metabolites in body fluids for predicting/detecting HCC development. Using a mass spectrometry-based approach, Wang and colleagues profiled the serum metabolites in patients with HCC, liver cirrhosis, and normal livers [172]. The metabolite canavaninosuccinate was significantly increased in the serum of patients with HCC, but decreased in patients with cirrhosis. Furthermore, canavaninosuccinate was able to predict HCC with a sensitivity of 80% and specificity of 100%, which is much higher than AFP. All patients in this study had HBV-associated HCC, and it will be important to investigate if the same metabolites are increased in HCC associated with other etiologies.

### 4.2. Prognostic Biomarkers

HCC tumor recurrence occurs in approximately 70% of patients treated with resection or ablative therapies [34]. This major clinical dilemma underscores the need for predictive markers to aid in patient selection and in guiding surveillance/adjuvant treatment (Figure 1). Pathological characteristics such as vascular invasion and multifocality are independent predictors of recurrence, but are difficult to evaluate preoperatively [35]. Serum biomarkers such as AFP, circulating DNA, and miRNAs are also being investigated for their predictive and prognostic potential [174,175]. Perhaps the most promising biomarkers for predicting recurrence are molecular signatures based on HCC tumors or the tumor microenvironment.

Gene expression profiling of resected HCC tumors has identified molecular signatures with prognostic potential. Over 20 gene signatures associated with HCC have been reported, including genes associated with survival [176], metastasis [36], and early recurrence [177]. The clinical utility of these signatures is controversial as their prognostic power has yet to be validated in large studies [35]. Nault *et al.*, published a five-gene score based on the expression of HN1, RAN, RAMP3, KRT19, and TAF9 that could predict prognosis (overall survival, early tumor recurrence, and risk of death after recurrence) in patients after resection more accurately than previously reported molecular signatures [44]. The authors also developed a “clinico-molecular” nomogram combining the five-gene score, Barcelona clinic liver cancer (BCLC) classification, and microvascular invasion to stratify patients based on low, medium, and high rates of recurrence. While still needing validation in prospective studies, the five-gene score and nomogram might be applicable in selecting candidates for liver transplantation. For instance, if a patient is outside of Milan criteria but the tumor has a low risk five-gene score, consideration of a liver transplant may still be warranted and *vice versa*.

One-third of HCC recurrences will occur more than two years after surgical resection [178]. Such late recurrences are considered to be *de novo* tumors rather than metastasis from the primary tumor [179]. Molecular signatures derived from the surrounding non-tumor cirrhotic tissue instead of the primary tumor can predict late recurrence. Budhu *et al.* used non-cancerous hepatic tissue from patients with venous metastasis to identify a 17-gene signature enriched in immune and inflammatory response genes [180]. This signature could predict metastasis, overall survival, and tumor recurrence more accurately than clinical parameters such as microvascular invasion and the Child-Pugh score. However, the 17-gene signature was derived from patients only with HBV-associated HCC tissue. Using tissues from patients with different HCC-associated etiologies (HBV, HCV, and EtOH), Hoshida *et al.* reported a 186-gene poor-survival signature enriched in adjacent tumor tissue which can predict overall survival and recurrence [179]. The question then arises as to which tissue should be subjected to molecular profiling to predict recurrence. Perhaps a combination of both: profiling the tumor might be best for predicting early recurrence, while profiling the adjacent non-tumor tissue could predict late recurrence [35].

### 4.3. New Therapeutic Strategies for HCC

Since HCC occurs in the background of diverse etiologies, a thorough understanding of the precise molecular factors driving the disease has been difficult. In a European cohort of 24 HCC tumors, genomic sequencing identified five to 121 mutations per tumor [6]. In addition to the heterogeneity in the number of mutations, there was no common mutation found in the majority of tumors. Identification of a common druggable molecular target, similar to BRAF^V600E^ in melanoma or BCR-ABL in chronic myelogenous leukemia, is unlikely in HCC. Nevertheless, subtypes of HCC are becoming apparent through large-scale genomic and transcriptomic sequencing. Targeting-altered cellular pathways in these subtypes may yield novel therapeutic strategies.

Whole genome and exome sequencing of ~400 tumors identified TERT, TP53, B-catenin, and ARID1A as the most frequently mutated genes in HCC [6,181,182,183,184]. The prevalence of some of these mutations is associated with etiology. TP53 mutations occur in >50% of HBV-related HCC, while B-catenin mutations are more frequent in the background of EtOH-associated HCC [6,181,182]. Common chromosomal gains are less prevalent but result in amplifications of cyclin D1 (11q13), FGF19 (11q13), VEGFA2 (6p21), Myc (8q), and Met (7q31) genes [185,186,187]. Chromosomal losses of CDKN2A (9p) and IGF2R (6p) have also been reported [188,189]. Functional classification of these mutated/amplified genes has found key pathways altered in HCC. Pathways include: Wnt/B-catenin, PI3K-AKT-mTOR, MAPK, telomere maintenance, cell cycle regulation, chromatin remodeling/epigenetic regulation, IGF signaling, and Il-6/JAK-STAT [174,181]. Evidence from mouse models of HCC have validated the functional importance of the Wnt/B-catenin pathway, and Myc, Met, and cyclin D1 genes [190,191,192,193]. Which of these pathways are oncogenic “drivers” and should be therapeutic targets for HCC remains unknown.

Sequencing has identified genomic alterations in HCC, but the question still remains as to how these molecular analyses can translate into effective therapeutic strategies (Table 4). One approach is to target mutationally activated pathways with specific pathway inhibitors. Since RAS mutations drive activation of the RAF-MAPK-MEK cascade, a trial is ongoing targeting RAS-mutated HCC with the MEK inhibitor, refametinib (NTC01915589) [80]. Many of the pathways activated in HCC such as the Wnt/B-catenin pathway or TP53 alterations are not currently considered druggable targets. An alternative approach is to use the pathway activation or molecular subgroup information as a biomarker to predict response. For instance, Finn *et al.* used the signatures published by Lee *et al.* to classify human HCC cell lines into subgroups, having either a hepatoblast (HB) or hepatocyte (HC) signature [176,194,195]. Interestingly, the HB cell lines were sensitive to the SRC/ABL tyrosine kinase inhibitor, dasatinib, while HC cell lines were not. Hence, one could hypothesize that patients with tumors having the HB signature would respond to dasatinib. Since specific genetic alterations are associated with different environmental exposures (EtOH, HBV), it will also be useful to determine if HCC cells respond to targeted therapeutics in an etiology-specific manner.

**Table 4:** Emerging therapeutic targeting approaches for HCC.

Targeting Approach	Molecular Alteration/Gene Signature (% Alteration Frequency)	Status of Therapeutic Targeting
Direct targeting of genetically altered genes in tumors (mutations or DNA amplifications)	KRAS/NRAS mutations (<5% [6,196,197])	Phase I RCT for HCC: refametinib (RAS-RAF-MEK pathway inhibitor) NTC01915589 [80]
	c-MET amplification (<5% [185,198])	Phase II RCT for HCC: tivantinib (c-Met inhibitor) [80]
Targeting of altered cellular pathways in tumors (based on genomic alterations and gene expression)	Wnt/B-catenin (B-catenin 18% [198,199], APC < 5% [6], AXIN < 15% [6])	LGK974 (Porcupine inhibitor ) in preclinical testing [200]
	Telomere maintenance (TERT 40% [184])	Antisense nucleotides targeting telomerase in preclinical testing [201]
Targeting of altered cellular pathways in tumors (based on genomic alterations and gene expression)	Chromatin remodeling (ARID1A < 20% [6,181,182], ARID2 < 10% [196,202], MLL complex < 15% [181,182])	Resminostat, vorinostat, belinostat (HDAC inhibitors) in CTs [203]
	PIK3-AKT-mTOR (PTEN < 5% [182], PIK3CA < 5% [6,196], RPS6KA3 ~10% [6,189])	Everolimus, sirolimus (mTOR) [174] in CTs for HCC, MK-2206 (AKT1) [204] in CTs for solid cancers
	IGF-signaling (phosphorylation of IGF-1R 20% [188])	Multiple CTs for HCC: Cixutumumab (IGF-1R ab) ± sorafenib, OSI-906 (IGF-1R inhibitor) [205]
	JAK-STAT signaling (JAK1 9%,Il-6R [181])	Ruxolitinib (JAK1/2) used for hematological malignancies [206]
	TP53 pathway/Cell cycle (TP [66] ~30% [6,174,183], RB < 10% [189])	Preclinical development
	Oxidative stress (NFE2L2 < 10% [6])	
Targeting tumor subtypes based on gene expression signatures	Hepatoblast/hepatocyte signature [176] Metastasis gene signature [36] Survival gene signature [179]	Preclinical testing. HCC cell lines with hepatoblast signature respond to dasatinib (Src/Abl inhibitor) [194]
Targeting tumors with immunotherapy	High expression of glypican-3 [207,208,209] Immune checkpoint blockade [210]	Some Glypican-3 antibodies in CTs [211]; JX-594 (oncolytic virus) targeting HCC cells in CT [212,213], anti-CTLA-4/PD-1 (immune checkpoint inhibitors ) in CTs [210]

Abbreviations: CT, clinical trials; HDAC, histone deacetylase inhibitor; ab, antibody.

Immunotherapy is emerging as a treatment approach for HCC (Table 4). Since the liver does not metabolize most immunotherapeutic drugs, they are appealing for patients with cirrhosis [212]. Promising immunotherapeutics for HCC include oncolytic viruses, CTLA-4 blockade, and tumor-antigen specific antibodies. The oncolytic virus JX-594 specifically infects and lyses tumor cells and expresses GM-CSF to stimulate an anti-tumor T cell response [212]. In a phase II trial of 30 patients with advanced HCC, an intra-tumoral injection of high dose JX-594 was associated with a longer median survival compared to low-dose JX-594 (14.1 *vs.* 6.7 months) [213]. CTLA-4 blockade can increase tumor-specific T cell activity by preventing T cell exhaustion. In patients with advanced HCV-associated HCC, the CTLA-4 antibody tremelimumab had a tolerable toxicity profile and was associated with a 76% disease control rate [210]. The surface glycoprotein, glypican-3 (GPC3) is overexpressed in more than 70% of HCC tumors and functions as a regulator of both Yap and Wnt signaling pathways [207,208,209]. Blocking GPC3 with an antibody was shown to be safe in patients with advanced HCC. In addition, patients with high-expressing GPC3 tumors *vs.* low-expressing GPC3 tumors had a prolonged time to progression (26 *vs.* 7.1 weeks) [211].

## 5. Conclusions

Selecting the optimal approach to target HCC is challenging. Reasons include aggressive tumor biology, presentation in the context of severe liver disease, and the lack of universally accepted treatment guidelines. As we have reviewed, the majority of clinical decisions are currently based on tumor size, number of lesions, and liver function. For patients with small tumors and preserved liver function, treatments of choice are either resection or PEI/RFA. In decompensated cirrhotics having tumors within the Milan criteria, liver transplantation is the best treatment modality. Large or multifocal tumors are usually targeted first with TACE and poor responders or those with severe liver impairment are offered sorafenib. While this clinical approach has proven effective in some cases—in particular using the Milan criteria for selecting transplant candidates—the dismal prognosis for most patients with HCC highlights the need for new therapeutics and better patient selection for established treatment options. Fulfilling this need is the emergence of nomograms, and protein, nucleic acid, and metabolic biomarkers, which can accurately predict prognosis in order to guide treatment choices, post-treatment surveillance, and adjuvant therapies. Furthermore, genomic analyses have stratified HCC tumors into subtypes, and determining if these subtypes respond uniquely to therapy will be a tremendous advance to guiding treatment decisions. Combination approaches (surgery and targeted therapy) or immunotherapy hold promise to reduce rates of recurrence or act as bridges to resection or transplantation. Overall, it is clear that treatment strategies for HCC are evolving, and we are optimistic HCC will eventually change from being a death sentence to a manageable disease.
